# SP, CGRP changes in pyridoxine induced neuropathic dogs with nerve growth factor gene therapy

**DOI:** 10.1186/s12868-015-0236-5

**Published:** 2016-01-05

**Authors:** Joo-Yeon Kang, Dae Young Yoo, Kwon-Young Lee, Wooseok Im, Manho Kim, Jung Hoon Choi, Hwa-Young Youn, Sae Hoon Kim, In Koo Hwang, Jin-Young Chung

**Affiliations:** Department of Veterinary Internal Medicine and Geriatrics, College of Veterinary Medicine, Kangwon National University, Hyoja2-dong, Chuncheon-si, Gangwondo 200-701 South Korea; Department of Anatomy and Cell Biology, College of Veterinary Medicine, and Research Institute for Veterinary Science, Seoul National University, Gwanak-ro 599, Gwanakgu, Seoul, 151-742 South Korea; Department of Anatomy, College of Veterinary Medicine, Kangwon National University, Hyoja2-dong, Chuncheon-si, Gangwondo 200-701 South Korea; Department of Neurology and Protein Metabolism Medical Research Center, College of Medicine, Seoul National University Hospital, 101 Daehakro, Chongno-ku, Seoul, 110-744 South Korea; Department of Veterinary Internal Medicine, College of Veterinary Medicine, Seoul National University, Gwanak-ro 599, Gwanakgu, Seoul, 151-742 South Korea; Department of Orthopedic Surgery, College of Medicine, Seoul National University Hospital, 101 Daehakro, Chongno-ku, Seoul, 110-744 South Korea

**Keywords:** Calcitonin gene-related peptide, Dog, H reflex, Nerve growth factor, Pyridoxine, Neuropathy

## Abstract

**Background:**

Nerve growth factor (NGF) is known not only as a major factor for neuronal plasticity but also as a pain stimulator. Although there have been several trials with NGF for its application in the regeneration or protection of the nervous system, the pain induced by NGF remains a challenge to be overcome. In this study, the pain induced by NGF gene therapy was evaluated.

**Results:**

Vehicle or recombinant dog NGF plasmid was administered into the intrathecal space of dogs. Twenty-four hours after the vehicle or NGF plasmid inoculation, dogs were subcutaneously treated with 150 mg/kg pyridoxine every day for 7 days. For pain assessment, physical examination and electrophysiological recording were performed. Only in the vehicle-treated group, weight loss occurred, while NGF plasmid inoculation significantly improved this physical abnormalities. In the vehicle-treated group, electrophysiological recordings showed that H-reflex disappeared at 24 h after the last pyridoxine treatment. However, in the NGF plasmid inoculated group, the H-reflex were normal. In the results of immunohistochemistry, the NGF plasmid administration efficiently expressed in the dorsal root ganglia and significantly increased the pyridoxine-induced reduction of calcitonin gene-related peptide (CGRP) immunoreactive neurons, but not in substance P immunoreactive neurons, in the dorsal root ganglia.

**Conclusions:**

Given these results, we reason that NGF gene therapy in pyridoxine induced neuropathic dogs does not induce neuropathic pain with this dosage, even with increasing the expression of CGRP.

**Electronic supplementary material:**

The online version of this article (doi:10.1186/s12868-015-0236-5) contains supplementary material, which is available to authorized users.

## Background

Neurotrophic factors are undoubted stimulators for the outgrowth of specific neuronal populations [1, 2]. Due to their potency, neurotrophic factors have drawn high expectations from scientists and various trials have attempted to investigate their efficacy [1]. Among the various neurotrophic factors, nerve growth factor (NGF) was the first neurotrophic factor discovered [3] and it was revealed that it impacts the development and maintenance of neurons in the peripheral nervous system and the functional integrity of neurons in the central nervous system [4]. For this reason, various preclinical characterizations and clinical trials with NGF have been performed [2]. In cases of clinical trials with recombinant human NGF (rhNGF) on peripheral neuropathies, phase I trials have shown that dose-dependent mild to moderate muscle pain and hyperalgesia appeared at the injection site as a side effect [5, 6]. Phase II trials of diabetic polyneuropathy indicate that rhNGF significantly improves the neuropathic symptoms, but produces dose-dependent hyperalgesia at the injection site [7, 8]. Other research has also demonstrated that although NGF develops and maintains the peripheral nervous system, it also contributes to producing hyperalgesia [9–11].

Although the evidence of neuropathic pain with NGF is obvious, there are still trials for NGF treatment because it has great pharmacological potential for the treatment of central neurodegenerative diseases and for peripheral neuropathies [2]. Interestingly, although it has been demonstrated that dose-dependent hyperalgesia is present at the injection site after a recombinant human NGF subcutaneous injection for peripheral neuropathies [8], there were no observable side effects when small amounts of NGF were injected through an intra-cerebro-ventricular channel for central nervous system diseases [12]. However, when injections of large amounts of NGF were administered, weight loss or back pain was reported even though it was administrated intra-cerebro-ventricularly [13]. Based on these results, we could understand that the route of injection and the amount of NGF being injected are important factors for the occurrence of neuropathic pain.

Hyperalgesia is a significant symptom for patients with neuropathic pain. Neuropathic pain is initiated or caused by a primary lesion to, or dysfunction in the nervous system [14]. Neuropathic pain and the dorsal root ganglion (DRG) are closely linked with each other. It is evident that hyperexcitability of DRG neurons is an important component for neuropathic pain [15]. The DRG neurons contain several kinds of peptides, which are related to various sensations [16]. Especially in the DRG, many small-diameter neurons are nociceptive and possess neuropeptides such as substance P (SP) and calcitonin gene-related peptide (CGRP), which are released in response to noxious simulation, are the main peptides for nociception [17]. It has been discovered that NGF-sensitive, tyrosine kinase receptor A (trkA) participates in pain processing and that neurons labeled with trkA overlap with neurons labeled with SP and CGRP [12, 13].

Sensory neuropathies, which is one of the peripheral neuropathies, are frequently associated with diabetes or anticancer therapies and less frequently associated with vitamin deficiency, hypothyroidism, uremia, and inherited metabolic disorders [18, 19]. Therefore, it is very important to develop animal models of sensory neuropathies. There are animal model studies using pyridoxine induced peripheral neuropathies [20, 21]. However these studies used extreme doses of pyridoxine or took a long period of time [22–24]. Chung et al. confirmed a dog model of sensory neuropathy by administering subcutaneous injections of pyridoxine over a short period of time [25]. This dog model fulfilled the requirement of advanced animal models with sensory neuropathies.

Based on these studies, we proposed a hypothesis that NGF gene therapy for pyridoxine induced neuropathy in dogs [26] could cause hyperalgesia. In this study, for identifying the relationship between NGF gene therapy and hyperalgesia, pain assessment including physical examination and electrophysiological recordings, and the changes in SP and CGRP levels were evaluated in dogs with pyridoxine-induced neuropathy that were administered NGF gene therapy.

## Results

### Pain assessment

#### Physical examination

**Fig. 1:**
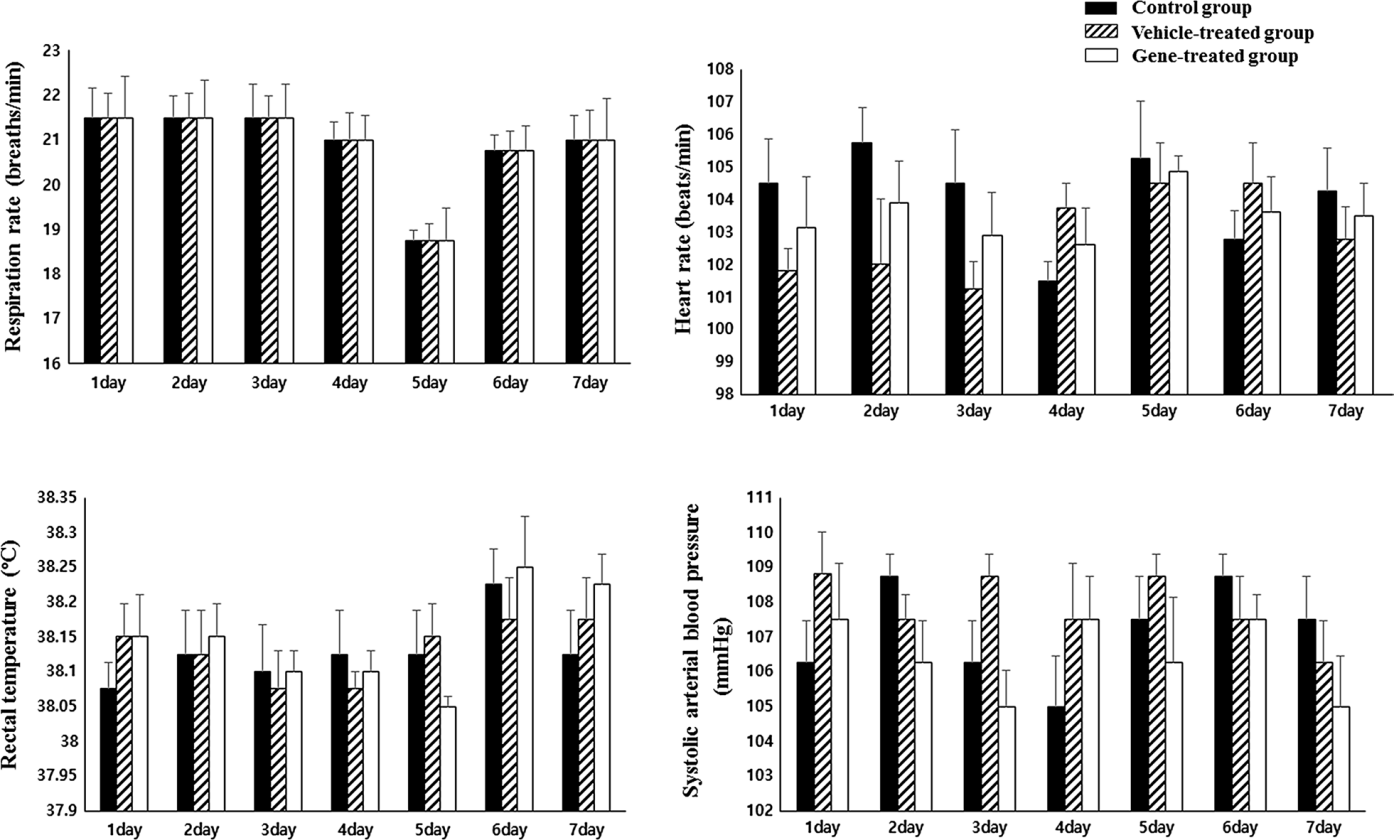
The respiration rate, heart rate, rectal temperature and systolic arterial blood pressure of all three groups were within normal ranges during the experimental period without significantly changes. This results showed that there is no evidence of tachypnea, tachycardia, hypertension, and hyperthermia in all group, which are general physiological indicators of pain (*n* = 4 per group)

**Fig. 2:**
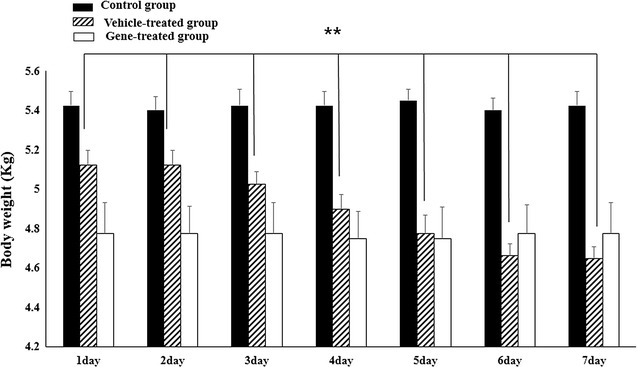
Body weight changes significantly only in the vehicle-treated group (p < 0.01) after pyridoxine treatment. This result evidences that NGF gene therapy in this study alleviated neuropathic pain, protecting the DRG from the neuropathic pain normally produced by pyridoxine (*n* = 4 per group)

The respiration rate (normal range 10–30 breaths/min), heart rate (normal range 70–180 breaths/min), rectal temperature (normal range 38.0–39.1 °C) and systolic arterial blood pressure (normal range 110–140 mmHg) of all three groups were within normal ranges during the experimental period (Fig. 1). However, weight measurements showed that there was significant weight loss only in the vehicle-treated group (p < 0.01), while there were no weight changes for the control group and the gene therapy group (Fig. 2).

#### Electrophysiological recording

**Fig. 3:**
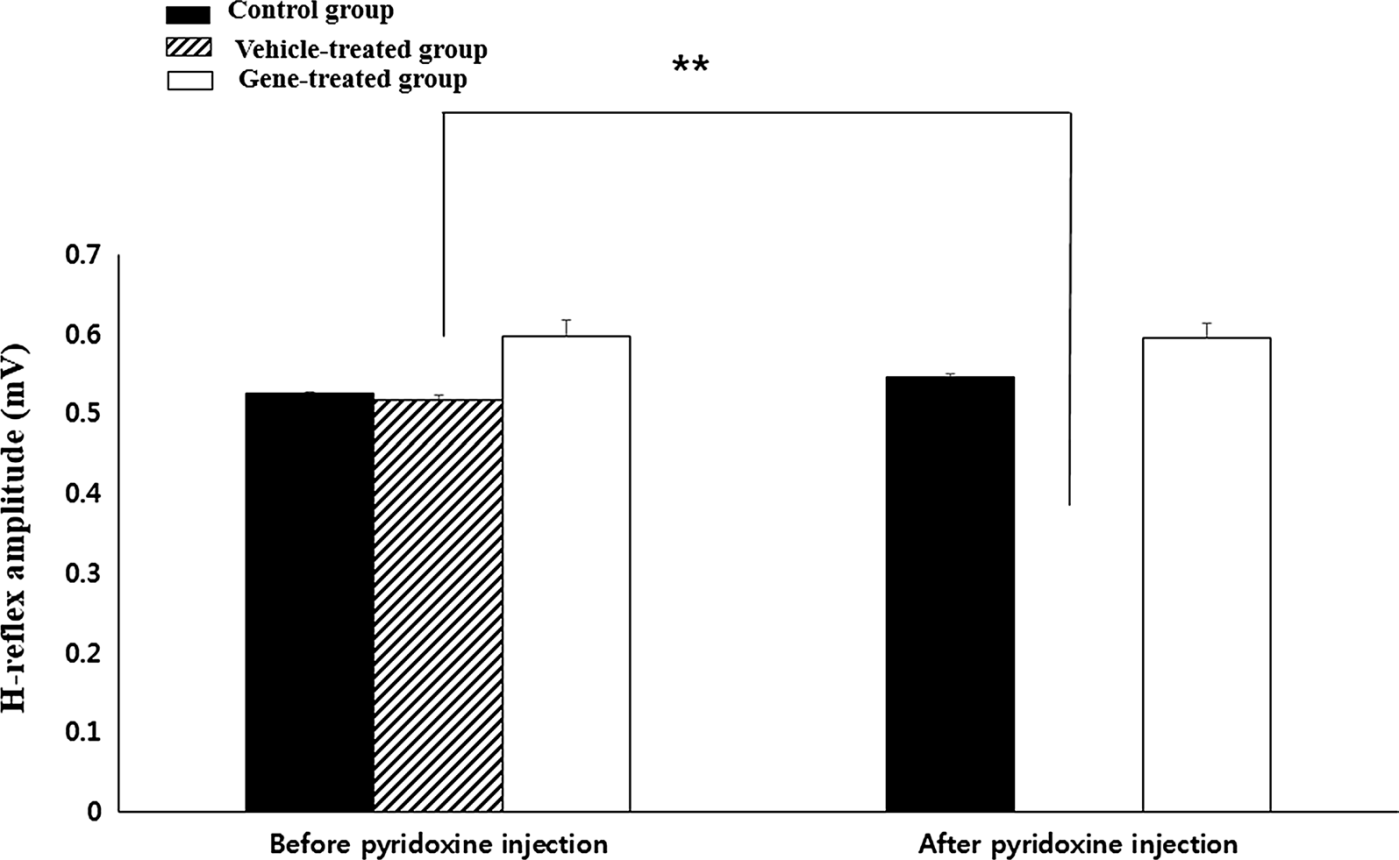
H-reflex disappears after the pyridoxine treatment in the vehicle-treated group (P < 0.01) however, H-reflexes do not change after the pyridoxine injection in the gene-treated group. This result evidences that NGF gene therapy in this study alleviated neuropathic pain (*n* = 4 per group)

Electrophysiological readings were recorded to measure M waves and H-reflexes in all groups before and after pyridoxine treatment. The M wave amplitude in all groups showed no remarkable change before and after the pyridoxine administration (*p* > 0.05). In the control group, H-reflexes did not change before or after pyridoxine injection. However, there was a remarkable change in the H-reflex before and after the pyridoxine treatment in the vehicle-treated group. Before pyridoxine injection, the average amplitude of the H-reflex was 0.52 ± 0.06 mV. After the pyridoxine injection, however, there was no consistently detectable H-reflex in the vehicle-treated group (*p* < 0.01). The H-reflexes in the NGF therapy group slightly decreased after pyridoxine injection, but significant differences were not detected before or after the pyridoxine injection (*p* > 0.05) (Fig. 3).

### Postural reaction assessments

**Fig. 4:**
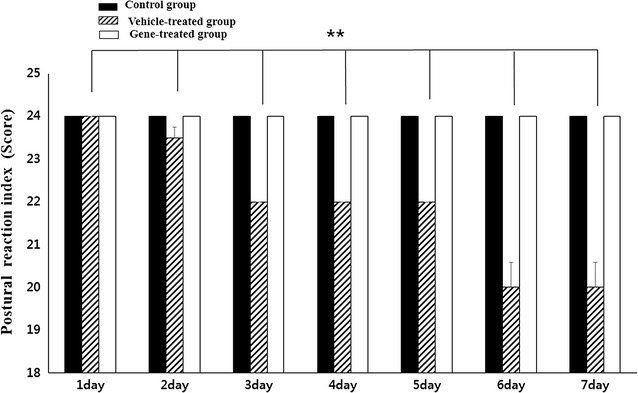
On the postural reaction assessment, all the dogs only in the vehicle-treated group developed a neurological disorder, most prominently in the hindquarters on the third day of the pyridoxine injections. These conditions were maintained until the end of the injections. In contrast, all of the dogs in the control group and the gene therapy group were normal during the postural reaction test (n = 4 per group; **P < 0.01)

On the postural reaction (hopping, wheelbarrowing, extensor postural thrust and proprioceptive positioning) assessment, all the dogs in the vehicle-treated group developed a neurological disorder, most prominently in the hindquarters. In addition, all dogs in this group started to show proprioceptive abnormalities involving their hindquarters as detected by the postural reaction test on the third day of the pyridoxine injections. These conditions were maintained until the end of the injections (*p* < 0.01). In contrast, all of the dogs in the control group and the gene therapy group were normal during the postural reaction test (Fig. 4).

### Histopathological analysis

#### Expression of NGF in the DRG

In the vehicle-treated group, NGF-positive neurons were barely detected in the DRG of L4, while in the pyridoxine-treated group, many NGF-positive neurons were detected in the DRG (Additional file 1: Figure S1).

#### Neuronal damage in the DRG

**Fig. 5:**
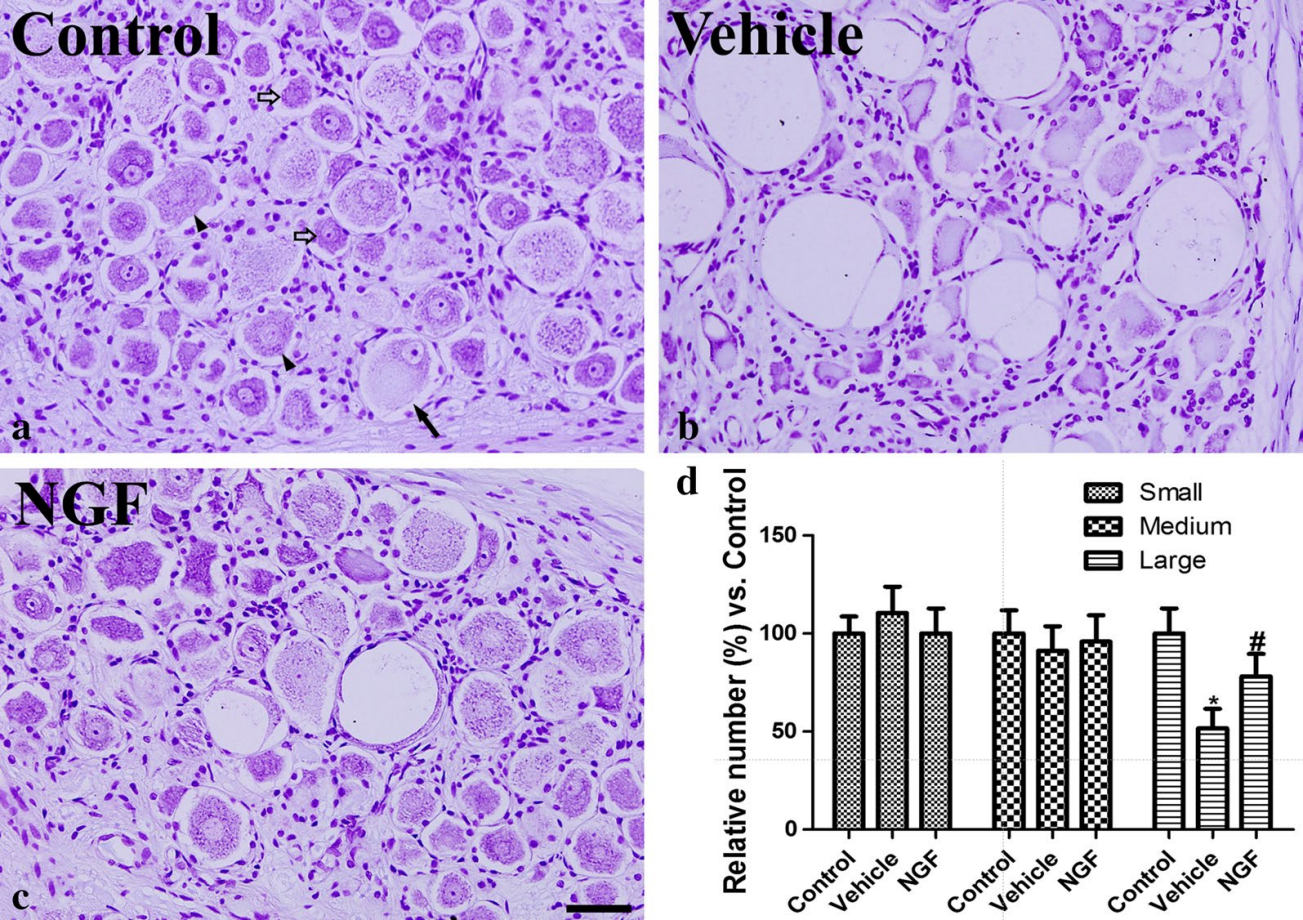
Cresyl violet staining of the dorsal root ganglion (DRG) in the control (**a**), vehicle-treated (Vehicle, **b**), and nerve growth factor gene-treated (NGF, **c**) groups at 4 weeks after pyridoxine injection for 1 week. In the control group, cresyl violet positive small- (*white arrow*), medium- (*arrow head*), and large-sized (*black arrow*) neurons are well detected in the DRG. Note that cresyl violet positive cells in the vehicle-treated group are few and some vacuoles are detected, while cresyl violet positive large-sized neurons are also abundant in the NGF group. *Scale bar* 100 μm. **d** Relative number as a % of small-, medium-, and large-sized neurons of the control group in the DRG (*n* = 4 per group; *P < 0.05, significantly different from the control group; ^#^ *P* < 0.05, significantly different from the vehicle group). The *bars* indicate the mean ± SEM

In the control group, cresyl violet positive neurons were abundantly detected in the DRG of L4 (Fig. 5a). In the vehicle-treated group, the presence of large-sized cresyl violet positive neurons was significantly decreased and those neurons were detected with vacuoles (Fig. 5b). In this group, the number of large-sized cresyl violet positive neurons was 51.7 % of the control group, while small- or medium-sized neurons were similarly detected compared to that in the control group (Fig. 5d). In the NGF therapy group, large-sized cresyl violet positive neurons were abundantly detected, although a few large vacuoles were also detected (Fig. 5c). In this group, the number of large-sized cresyl violet positive neurons was 78.1 % of the control group’s amount, while the number of medium- and small-sized neurons was not significantly different between them (Fig. 5d).

#### Immunoreactivity for SP and CGRP in the DRG

**Fig. 6:**
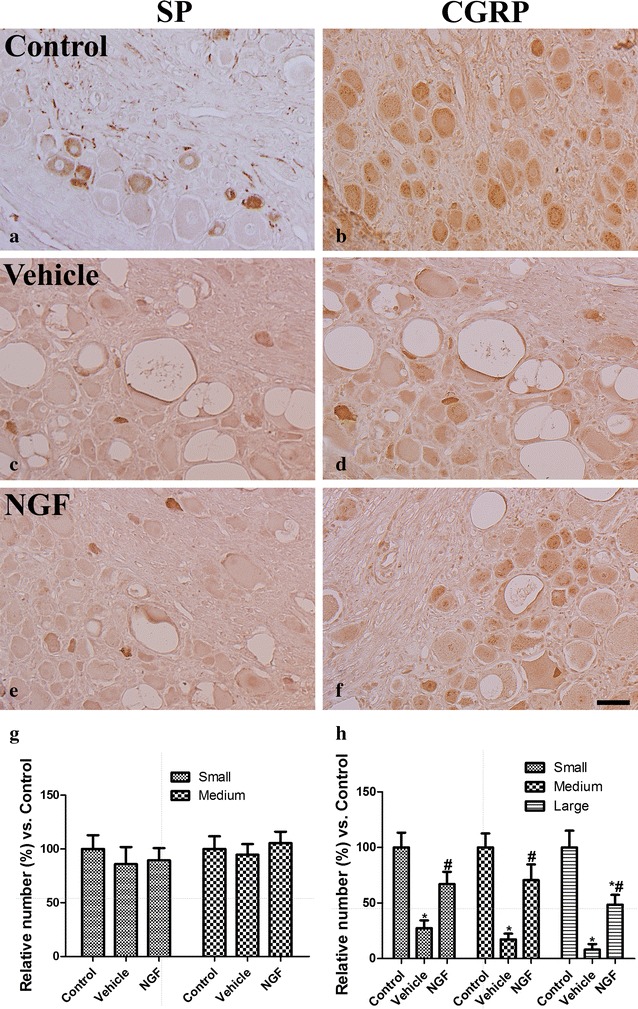
Substance P (*SP*) and calcitonin gene-related peptide (*CGRP*) immunostaining of the dorsal root ganglion (*DRG*) in the control (**a**, **b**), vehicle-treated (vehicle **c**, **d**), and nerve growth factor gene-treated (*NGF* **e**, **f**) groups at 4 weeks after 1 week of pyridoxine injection. In the control group, *SP* immunoreactive neurons are mainly detected in the small- and medium-sized neurons, while *CGRP* immunoreactive neurons in the small-, medium-, and large-sized neurons are well detected in the *DRG*. Number of *SP* immunoreactive neurons is similarly detected in all groups, while the number of *CGRP* immunoreactive neurons is decreased in the small- and medium-sized neurons of vehicle-treated group and this reduction is significantly ameliorated in the *NGF* group. *Scale bar* = 100 μm. **g** and **h**: Relative number as a % of small-, medium-, and large-sized neurons of the control group in the DRG (*n* = 4 per group; *P < 0.05, significantly different from the control group; ^#^ *P* < 0.05, significantly different from the vehicle group). The *bars* indicate the mean ± SEM

In the control group, SP immunoreactivity was mainly detected in the small- and medium-sized neurons, as well as the neuropil (Fig. 6a), while CGRP immunoreactivity was detected in all sized neurons (Fig. 6b). In the vehicle-treated group, the number of SP immunoreactive neurons was slightly decreased in the DRG compared to that in the control group, but the difference between the groups did not achieve significance (Fig. 6c, g). However, the number of CGRP immunoreactive small-, medium-, and large-sized neurons in the vehicle-treated group was significantly decreased compared to that in the control group (Fig. 6d, h). For the NGF-therapy group, SP immunoreactive neuron detection was similar to the vehicle-treated group (Fig. 6e, g). In contrast, the number of CGRP immunoreactive medium- and small-sized neurons was significantly greater in the DRG of the therapy group than that in the vehicle-treated group (Fig. 6f, h).

## Discussion

A previous study found a protective effect of NGF gene therapy against pyridoxine induced sensory neuropathy in a dog model [26]. In this study, the characteristics of the pain induced by NGF gene therapy were evaluated in the same model. In the present study, we observed the significant reduction in the number of large-sized neurons in the DRG after pyridoxine intoxication. This result was supported by previous studies that the administration of pyridoxine selectively causes the damage in the large-diameter A-cells of the DRG with larger-diameter myelinated fibers in the sciatic nerve [20, 27, 28]. In our studies, we also observed the vulnerability of large-sized neurons after pyridoxine intoxication in DRG of dogs [25, 26, 29, 30].

The biological effects of neurotrophins are mediated via two major receptors, tyrosine kinase (Trk) receptors and 75 kDa (p75NTR). Each neurotrophin binds with high affinity to a specific Trk receptor. In the case of NGF, it has a high affinity to TrkA and a lower affinity to p75NTR [31]. Both NGF-trkA signaling and NGF-p75NTR participate in the neuropathic pain pathway [32]. NGF-trkA signaling dynamically regulates the synthesis of nociceptive neuropeptides like SP and CGRP, which contribute to the sensation of neuropathic pain [33]. For the relief of pain, there is critical need for drugs other than non-steroidal anti-inflammatory drugs. Based on these concepts, there are various trials of NGF antibody which sought to block the neuropathic pain, with the results indicating NGF antibody as s possible painkiller [29, 30].

For diagnosis and treatment of neuropathic pain, questionnaires to determine whether the symptoms indicate neuropathic pain or not are required, so many kinds of questionnaires have been developed for humans [34]. There are many trials for scaling pain in animals; however, there are not any special scales for neuropathic pain [35–37]. General physiological indicators of pain are tachypnea, tachycardia, hypertension, and hyperthermia. Additionally, anorexia resulting in weight loss is common in animals with significant acute or chronic pain [38]. In this study, there were no changes of respiratory rate, heart rate, body temperature or systolic arterial blood pressure in all three groups. However significant weight loss was confirmed only in the positive control group, not in the gene therapy group. This result evidences that NGF gene therapy in this study mitigated neuropathic pain, protecting the DRG from the neuropathic pain normally produced by pyridoxine. In this study, the route of the intrathecal region was chosen and the single dose of 40 μg NGF was used in the dog model. The dosage was decided by previous papers which used same manufactural cationic polymer transfection reagent to the intrathecal region [39, 40]. Based on the immunohistochemistry for NGF, we found that NGF effectively expressed in the DRG, which is the damaged area by pyridoxine. We could assume that the amount of NGF was not enough to cause neuropathic pain, but just enough to protect the DRG from the pyridoxine-induced neuropathy.

Unfortunately, though patients with neuropathic pain have the same predisposing condition, their responses to it could be varied [41]. For the objectification of analyzing neuropathic pain, the development of diagnostic techniques to identify it is necessary. There are a few trials to objectify the pain using the H-reflex. The muscle potential is the resultant activity of a true monosynaptic reflex arc and thus is appropriately referred to as an H-reflex. It has been demonstrated that a decrease in the mean H-reflex amplitude was observed in patients with back pain [42, 43]. Similarly to those studies, it was also confirmed that the H-reflex disappeared only in the positive control group in this study.

Forty percent of the DRG neurons are large-sized, which give rise to myelinated axons. In the present study, we observed a reduction in the number of large-sized neurons in the DRG after pyridoxine injection. This result was bolstered by our previous study where pyridoxine injection was found to significantly reduce the myelinated fibers in the sciatic nerve [25, 26]. NGF therapy is shown to significantly prevent the pyridoxine-induced decreases in the number of large-sized neurons in the DRG. The other 60 % of medium-/small sized neurons can be divided into neuropeptidergic and nonpeptidergic groups. The neuropeptidergic DRG neurons are easily detected by the CGRP antibody, comprising mostly small neurons with unmyelinated axons (C fibers) and innervating mainly polymodal nociceptors [44]. SP and CGRP are also expressed by a group of medium-sized cells with finely myelinated (Aδ) axons, most of which are nociceptors of the high-threshold mechanoreceptor type [44]. CGRP has important roles in trophic effects in the regeneration of peripheral nerves [45] except nociception. In this study, the significant reduction of CGRP may be associated with impairments of these trophic functions. This result was supported by previous study that the number of CGRP-positive neurons was significantly decreased in type 1 and 2 diabetic rats compared to their controls [46].

## Conclusion

These results suggests that NGF gene therapy protects large-sized neurons from pyridoxine-induced injection and helps maintain tropic actions in the regeneration of peripheral nerves connected to DRG. With this study, we conclude that NGF gene therapy in pyridoxine induced neuropathic dogs does not induce neuropathic pain and facilitates the regeneration of pyridoxine-induced neuropathies in the DRG.

## Methods

### Animal model

Twelve dogs were used in this experiment, comprising 6 males and 6 females, each around 2 years of age. Their body weights ranged from 4 to 6 kg. All dogs were clinically judged to be in good health and neurologically normal. The handling and care of the animals was in compliance with current international laws and policies (NIH Guide for the Care and Use of Laboratory Animals, NIH Publication No. 85-23, 1985, revised 1996) and were approved by the Institutional Animal Care and Use Committee (IACUC) of Seoul National University (Approval no.: SNU-060623-1). Dogs were divided into 3 groups; control, vehicle-treated and gene therapy group (*n* = 4 in each group). To induce neuropathy, pyridoxine (Sigma, St. Louis, MO, USA) was prepared in physiological saline (100 mg/mL) immediately before injection, and administered at 150 mg/kg subcutaneously, once a day in the morning for 7 days [25] to the vehicle-treated and gene therapy groups.

### Recombinant dog NGF gene therapy

Twenty-four hours before pyridoxine injection, all of the dogs were anesthetized with Zoletil 50^®^ (Virbac, Carros, France). Thereafter, depending on the groups to which the dogs belonged, a 400 μL vehicle (cationic polymer transfection reagent, Polyplus transfection, France) or constructed recombinant dog NGF plasmid solution (40 μg/400 μL) was administered through the intrathecal region using a 27-gauge needle. The constructed recombinant dog NGF plasmid was established in the previous study [26].

### Physical examination and postural reaction assessments

Physical examination including respiration rate (normal range 10–30 breaths/min), heart rate (normal range 70–180 beats/min), rectal temperature (normal range 38.0–39.1 °C), systolic arterial blood pressure (normal range: 110 mmHg -140 mmHg) and body weight of the test dogs were measured every morning during the test period. Postural reaction (hopping, wheelbarrowing, extensor postural thrust and proprioceptive positioning) assessments were also done on all dogs every morning during the test period (Table 1 in Appendix) [47].

**Table 1:** Postural reaction index

	Front		Rear	
	Right	Left	Right	Left
Hopping	ⓞ①②③④	ⓞ①②③④	ⓞⓞ①②③④	ⓞ①②③④
Wheelbarrowing	ⓞ①②③④	ⓞ①②③④		
Extensor postural thrust			ⓞ①②③④	ⓞ①②③④
Proprioceptive reaction	ⓞ①②③④	ⓞ①②③④	ⓞ①②③④	ⓞ①②③④

ⓞ Absent, ① Depressed, ② Normal, ③ Hyperactive, ④ Hyperactive with clonus

### Electrophysiological recording

At 24 h after the last pyridoxine treatment, all dogs were preanesthetized with atropine (0.1 mg/kg of body weight, IM). Anesthesia was induced with diazepam and was maintained with isoflurane through a semi-closed system. Subcutaneous temperature was maintained at 37–38 °C and Neuropack2 (Nihon Kohden, Japan) was used for all recordings. All measurements were performed in the left hindlimb and M waves were recorded from the tibial nerve, using a 1 Hz, 0.5 ms, supramaximal stimulus. Stimulating electrodes were positioned at the distal tibial nerve and the recording electrode was positioned at the plantar interosseous muscle. The ground electrode was positioned between the stimulating electrode and the recording electrode. The recording electrode was a bipolar needle electrode. The Hoffman (H)-reflex was recorded using a 1 Hz, 0.5 ms, submaximal stimulus. The stimulating electrode was positioned at the tibial nerve adjacent to the hook, with the recording and ground electrodes positioned at the same sites of the tibial nerve where M waves were measured. All measurements were performed at least eight times. Electrophysiological recordings were performed twice, once before the experiment and once after the test period.

### Tissue processing

For histological analysis, the dogs were anesthetized with a high dose of Zoletil 50^®^ (Virbac, Carros, France) and propofol (Myungmoon Pharm., Seoul, Korea), and perfused transcardially with 0.1 M phosphate-buffered saline (PBS), followed by 4 % paraformaldehyde in 0.1 M PBS to induce euthanasia at 4 days after the last pyridoxine treatment. After perfusion, tissues from the left and right DRG of L4 were quickly removed and post-fixed for 24 h in the same fixative at 4 °C. They were then dehydrated with graded concentrations of alcohol before being embedded in paraffin. Paraffin-embedded tissues were serially sectioned using a microtome (Leica Microsystems GmbH, Wetzlar, Germany) into 3-μm sections, then were mounted onto silane-coated slides (Muto Pure Chemicals Co., Ltd, Tokyo, Japan). The sections apart from 150-μm each other were stained with cresyl violet according to the standard protocol.

### Immunohistochemistry

The sections apart from 150-μm each other in all groups were hydrated and treated with 0.3 % hydrogen peroxide (H_2_O_2_) in PBS for 30 min. For antigen retrieval, the sections were placed in 400-mL jars filled with citrate buffer (pH 6.0) and heated in a microwave oven (Optiquick Compact, Moulinex) operating at a frequency of 2.45 GHz and an 800-W power setting. After three heating cycles of 5 min each, slides were allowed to cool to room temperature and were washed in PBS. After washing, the sections were incubated in 10 % normal goat serum in PBS for 30 min. They were then incubated with rabbit-NGF antibody (1:500; Biorbyt, San Francisco, CA, USA), rabbit anti-SP antibody (1:10,000; ImmunoStar, Hudson, WI, USA) or mouse-anti-CGRP (1:1,000; Abcam, Cambridge, UK) for 48 h at 4 °C. They were then exposed to either a biotinylated goat anti-rabbit IgG or anti-mouse IgG streptavidin peroxidase complex (diluted 1:200, Vector Laboratories, Inc., Burlingame, CA, USA), and visualized with 3,3ʹ-diaminobenzidine tetrahydrochloride (Sigma) in 0.1 M Tris–HCl buffer (pH 7.4).

The number of SP- or CGRP-immunoreactive neurons in each group of sections was counted in the DRGs using an image analyzing system equipped with a computer-based CCD camera (software: Optimas 6.5, CyberMetrics, Scottsdale, AZ, USA). DRG neurons were separated into three categories according to their sizes: small- (area 1000 μm^2^), medium- (area 1000–2000 μm^2^), and large-sized (>2000 μm^2^) neurons. The number of these neurons in all the groups was counted in the DRG using an image analyzing system equipped with a computer-based CCD camera (software: Optimas 6.5, CyberMetrics, Scottsdale, AZ). Cell counts were obtained by averaging the counts from DRG per section (10 sections) under a light microscope with a 10× lens.

## Statistical analysis

A repeated measure one-way ANOVA test was done for the analysis of body weight measurements during the experimental period and a *t* test was done for the electrophysiological recordings and histochemical results before and after the pharmacologic treatment. The level of significance was set at *p* < 0.05.

## Appendix

See Table 1.

## Additional file

10.1186/s12868-015-0236-5 Nerve growth factor (NGF) immunostaining of the dorsal root ganglion (DRG) in the vehicle-treated (A), and NGF gene-treated (B) groups at 4 weeks after 1 week of pyridoxine injection. In the vehicle-treated group, NGF immunoreactive neurons are not detectable in the DRG. In the NGF gene-treated group, NGF immunoreactive neurons are abundantly observed in the DRG. Scale bar = 50 μm.

## Abbreviations

CGRP: calcitonin gene-related peptide
DRG: dorsal root ganglion
H reflex: Hoffman-reflex
NGF: nerve growth factor
rhNGF: recombinant human NGF
SP: substance P
trk: tyrosine kinase receptor
