# Supplementary figures and images for: SP, CGRP changes in pyridoxine induced neuropathic dogs with nerve growth factor gene therapy
Source: BMC Neurosci. 2016 Jan 5;17:1. doi: 10.1186/s12868-015-0236-5 (PMC4700743; doi:10.1186/s12868-015-0236-5)

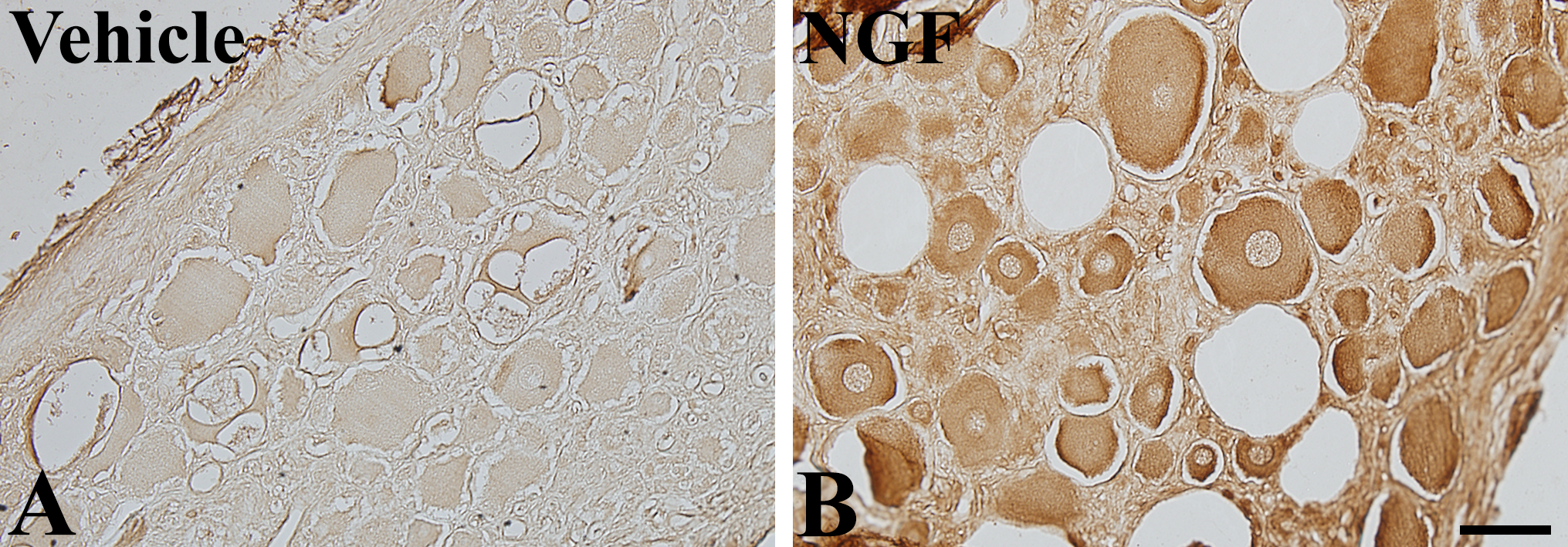

Supplement: Supplementary file 1 — 10.1186/s12868-015-0236-5 Nerve growth factor (NGF) immunostaining of the dorsal root ganglion (DRG) in the vehicle-treated (A), and NGF gene-treated (B) groups at 4 weeks after 1 week of pyridoxine injection. In the vehicle-treated group, NGF immunoreactive neurons are not detectable in the DRG. In the NGF gene-treated group, NGF immunoreactive neurons are abundantly observed in the DRG. Scale bar = 50 μm. [file 12868_2015_236_MOESM1_ESM.tif]
